# The HPV-18 E7 CKII phospho acceptor site is required for maintaining the transformed phenotype of cervical tumour-derived cells

**DOI:** 10.1371/journal.ppat.1007769

**Published:** 2019-05-22

**Authors:** Om Basukala, Suruchi Mittal, Paola Massimi, Marco Bestagno, Lawrence Banks

**Affiliations:** International Centre for Genetic Engineering and Biotechnology, Trieste, Italy; University of Wisconsin-Madison, UNITED STATES

## Abstract

The Human Papillomavirus E7 oncoprotein plays an essential role in the development and maintenance of malignancy, which it achieves through targeting a number of critical cell control pathways. An important element in the ability of E7 to contribute towards cell transformation is the presence of a Casein Kinase II phospho-acceptor site within the CR2 domain of the protein. Phosphorylation is believed to enhance E7 interaction with a number of different cellular target proteins, and thereby increase the ability of E7 to enhance cell proliferation and induce malignancy. However, there is little information on how important this site in E7 is, once the tumour cells have become fully transformed. In this study, we have performed genome editing of the HPV-18 E7 CKII recognition site in C4-1 cervical tumour-derived cells. We first show that mutation of HPV18 E7 S32/S34 to A32/A34 abolishes CKII phosphorylation of E7, and subsequently we have isolated C4-1 clones containing these mutations in E7. The cells continue to proliferate, but are somewhat more slow-growing than wild type cells, reach lower saturation densities, and are also more susceptible to low nutrient conditions. These cells are severely defective in matrigel invasion assays, partly due to downregulation of matrix metalloproteases (MMPs). Mechanistically, we find that phosphorylation of E7 plays a direct role in the ability of E7 to activate AKT signaling, which in turn is required for optimal levels of MMP secretion. These results demonstrate that the E7 CKII phospho-acceptor site thus continues to play an important role for E7’s activity in cells derived from cervical cancers, and suggests that blocking this activity of E7 could be expected to have therapeutic potential.

## Author summary

In this study we have used genome editing to mutate the HPV-18 E7 CKII phospho-acceptor site in cells derived from a cervical cancer. We demonstrate that this results in a decrease in cell proliferation and renders the cells particularly susceptible to low nutrient conditions. Furthermore these cells are defective in invasive potential and this appears linked to a decrease in the levels of secreted MMPs. Mechanistically this is linked directly to a role of the E7 CKII phospho-acceptor site in upregulating AKT signaling. These studies demonstrate that the E7 CKII site plays a direct role in maintaining a fully transformed phenotype, and indicates a novel function for this region of E7 in regulating AKT and the levels of secreted MMPs.

## Introduction

Human papillomaviruses (HPVs) are major causes of human cancer, with cervical cancer being the most important. Whilst there are over 200 different HPV types, only a small subset are responsible for the development of human cancers and, of these, HPV-16 and HPV-18 are the most common [1]. HPVs replicate in differentiating epithelia, in cells that would normally have exited the cell cycle. Since HPVs do not encode any proteins that can be used to replicate DNA, they need to drive these non-dividing cells back into cell cycle, so that the viral DNA can be amplified. This is brought about by the action of the two viral oncoproteins, E6 and E7, which together create an environment favourable for viral DNA replication [2]. This is achieved primarily through interfering with cellular growth control pathways, with E7 targeting many elements involved in the control of cell cycle, whilst E6 inhibits the pro-apoptotic response of the cell to this unscheduled DNA replication [3, 4]. In rare instances, the viral life cycle is perturbed and the events that, ultimately, give rise to malignancy are initiated. In these tumour-derived cell lines, E6 and E7 continue to be expressed, and loss of expression of either brings about cessation of cell growth and the induction of apoptosis [5–7]. Therefore, both proteins are excellent targets for therapeutic intervention in HPV-induced malignancy.

HPV E7 is a highly multifunctional protein. Major targets include the pRb family of tumour suppressors, which E7, by recruitment of cullin ubiquitin ligases, can target for proteasome-mediated degradation [8]. E7 also interacts with a large number of transcriptional regulators and other potential tumour suppressor proteins, such as PTPN14 [9, 10], and it has been shown to also recruit additional components of the ubiquitin proteasome pathway, including the UBR4/p600 ubiquitin ligase, amongst others [11, 12]. HPV E7 is also subject to phosphorylation by Casein Kinase II (CKII) [13, 14], and this appears to play an important part in the ability of E7 to bring about cell transformation [15]. At a molecular level this phosphorylation event is also thought to increase the interaction of E7 with a number of cellular target proteins, including pRb and TBP [16–19]. Indeed, recent studies identified a variant HPV-16 E7 with an extra CKII phospho-acceptor site, and this appeared to correlate with increased transforming potential [20]. Finally, recent structural studies have also begun to shed light on how phosphorylation of E7 might affect its overall structure, and thereby also affect target recognition [21].

Whilst it is clear that CKII phosphorylation of E7 plays an important role in the transformation of cells *in vitro*, there is little information as to how important this phospho-acceptor site is, once the cells have become fully transformed, and whether it plays any role in the maintenance of the transformed phenotype. Complementation assays using various E7 mutants, in cells where E6/E7 expression is suppressed by E2 overexpression, point to a role for CKII phosphorylation in optimal inactivation of the pRb pathway [22]. However, we have been interested in determining whether specifically blocking E7 phosphorylation by CKII in its true physiological setting could be considered as a potential option for therapeutic intervention in HPV-induced cancer. Therefore, in this study we have used a genome-editing approach to mutate the CKII phospho-acceptor site in HPV-18 E7 in cells derived from a cervical cancer. We show that these cells have profound defects in cell proliferation and invasion, which is accompanied by a marked decrease in the levels of expression of certain MMPs. These results demonstrate that phosphorylation of E7 by CKII is required for maintaining a fully-transformed phenotype, and identifies this as a potential route for therapeutic intervention in HPV-induced malignancy.

## Results

**Fig 1:**
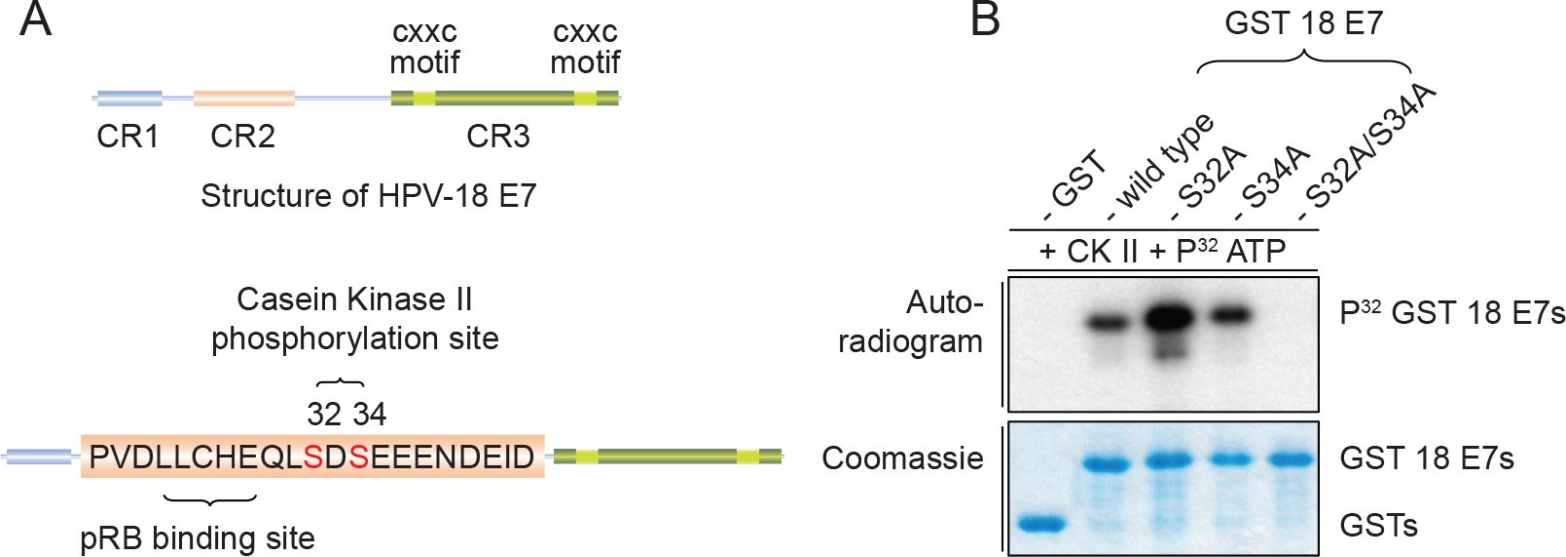
Mutation of HPV-18 E7 wild type to S32A/S34A abolishes phosphorylation by casein kinase II (CKII). **(A)** Schematic representation of HPV-18 E7 conserved regions (CR), the pRB binding site and CKII phosphorylation site in the CR2 domain. **(B)** *In vitro* phosphorylation of GST HPV-18 E7 fusion proteins. Purified GST 18E7 wild type and mutants, as indicated, were incubated with CKII and 32P-γATP. After extensive washing, bound proteins were subjected to SDS-PAGE and autoradiographic analysis. The upper panel represents the autoradiogram and the lower panel represents the Coomassie Blue-stained gel.

Previous studies have shown that HPV-16 E7 is phosphorylated at residues S31 and S32 by CKII, and protein alignments would also suggest that the equivalent residues S32 and S34 in HPV-18 E7 would likewise be similarly phosphorylated by CKII [14]. In order to confirm this, we first performed a series of *in vitro* phosphorylation assays on purified GST-18 E7, using commercially available purified CKII and ^32^P-γATP. To verify the identity of the phospho-acceptor site, we also generated single and double amino acid substitutions within the putative CKII phospho-acceptor site of HPV-18 E7. The results obtained are shown in Fig 1 and demonstrate that both S32 and S34 are phosphorylated by CKII. Interestingly, the single S32A mutation appears to increase the overall level of E7 phosphorylation, although the reasons for this are unclear. Mutation of both of these residues completely abolishes CKII phosphorylation of E7, confirming that these are the only CKII phospho-acceptor sites in HPV-18 E7. Taken together, these results demonstrate that both S32 and S34 are phosphorylated by CKII.

**Fig 2:**
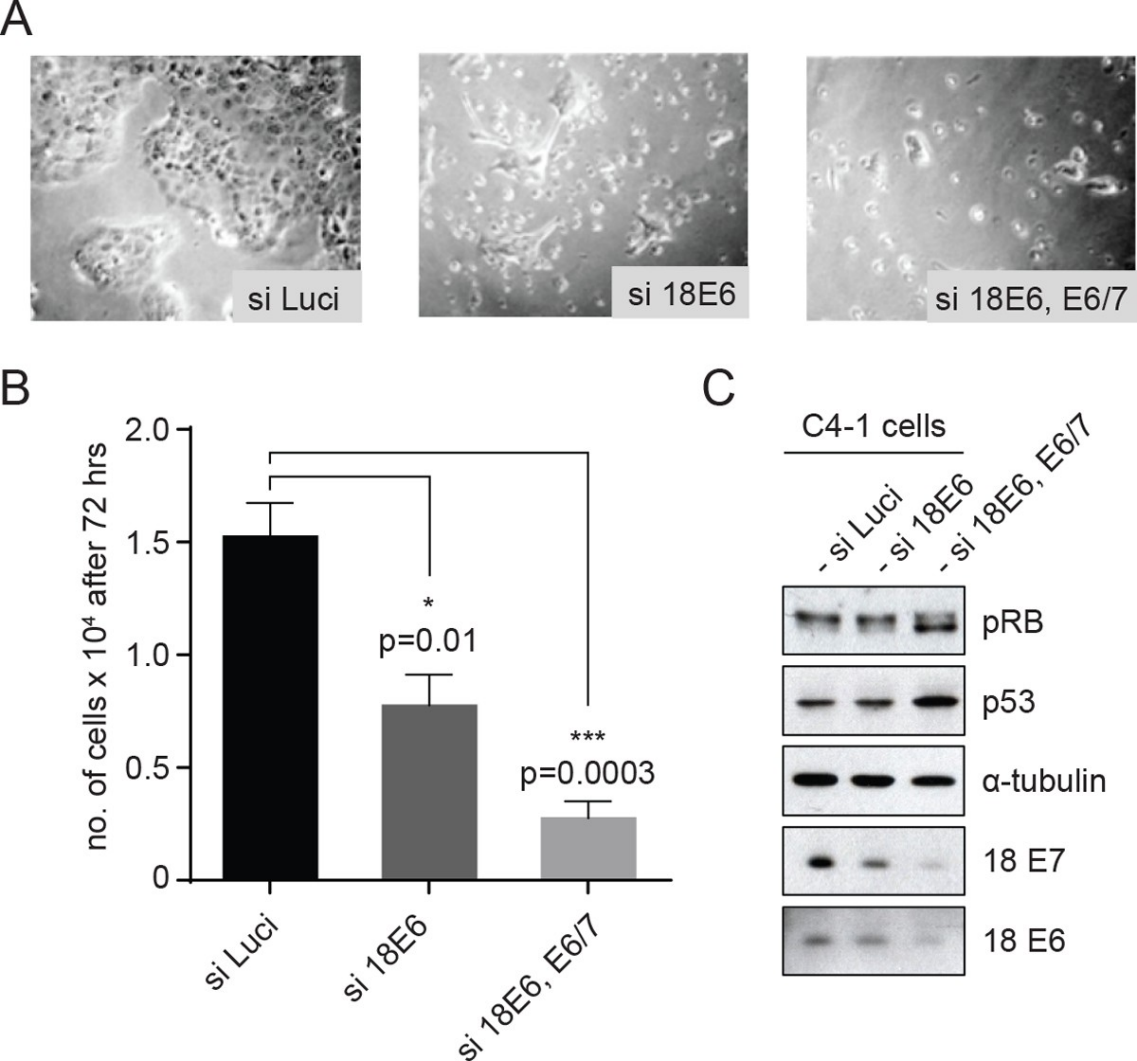
C4-1 cells are dependent on continued expression of E6 and E7 for growth and proliferation. **(A)** Micrograph of the cells after 72h of siRNA treatment. C4-1 cells were transfected with si-Luciferase (si-Luci), si-HPV18 E6, si-HPV18E6 and E6/7 together, using RNAimax transfection reagent. **(B)** After 72h incubation, cells were counted to assess the remaining cell number and the graph shows the quantification after the different siRNA transfections. Asterisks indicate the p-value (*—p<0.01, ***—p<0.0003) of mean cell number in siRNA transfections compared with siLuci, using an unpaired t-test. **(C)** Western blots showing levels of pRB, p53, α-tubulin, 18E7 and 18E6 after transfection with the indicated siRNAs.

We then proceeded to design strategies for mutating these residues in cells derived from a cervical cancer. To do this we chose C4-1 cells, which only have one copy of HPV-18 DNA integrated into the host genome, and are therefore more amenable to genome editing than a line containing multiple copies of integrated HPV genomes [23]. Prior to performing genome editing it was first necessary to confirm that C4-1 cells were indeed still dependent upon the continued expression of E6 and E7 for maintenance of the transformed phenotype. To do this, the cells were transfected with siRNAs targeting HPV-18 E6 and 18 E6/E7, and after 72hrs the numbers of cells remaining were counted. The siRNA-transfected cells were also analysed for the levels of p53 and pRB using Western blotting. The results, together with the microscopic appearance of the cells, is shown in Fig 2, where it can be seen that C4-1 cells are indeed still dependent upon continued E6/E7 expression for maintenance of cell survival, in agreement with similar studies done in other HPV positive cervical tumour-derived cell lines [5–7].

**Fig 3:**
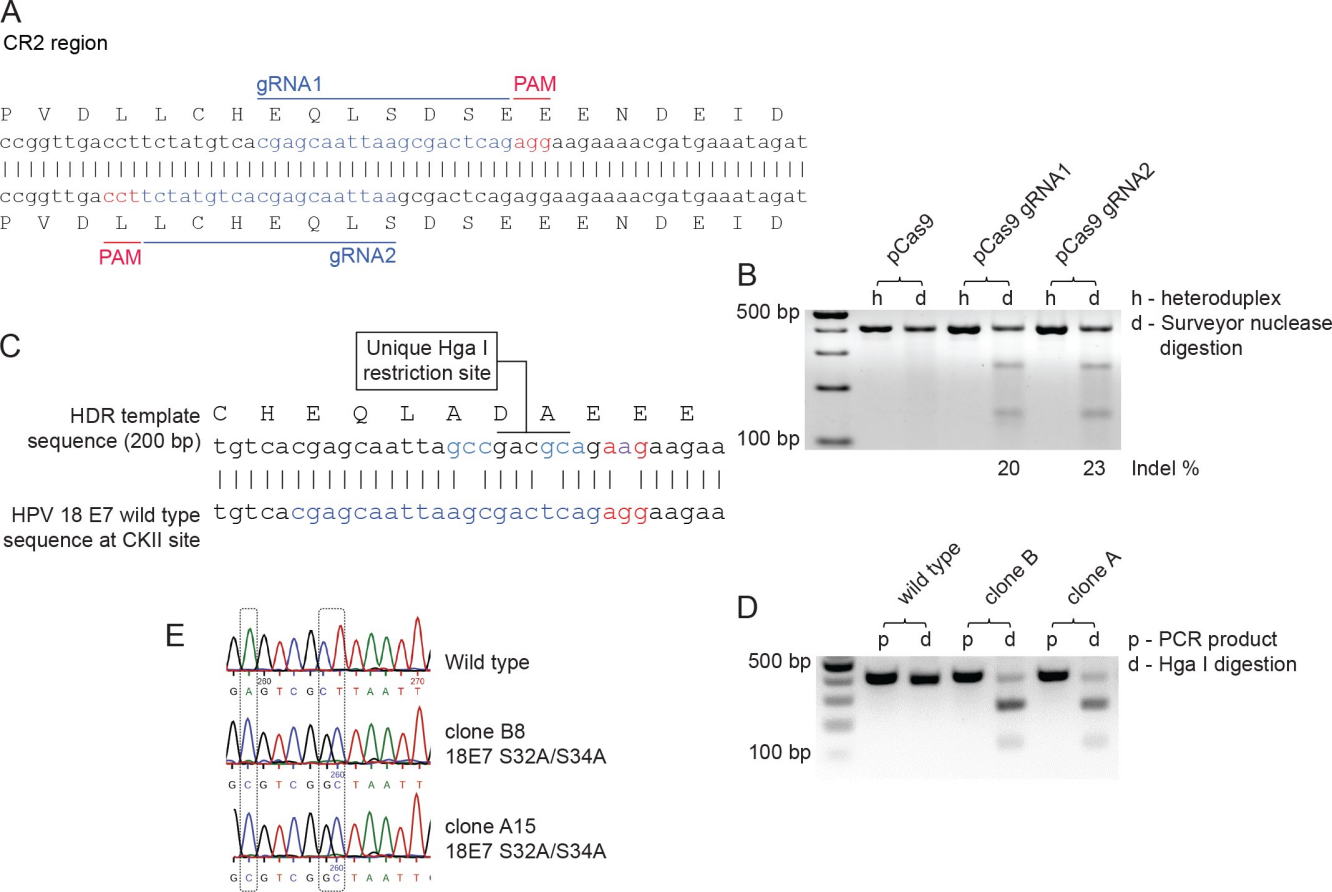
Strategy for genome editing HPV-18 E7 S32A/S34A in C4-1 cells. **(A)** Design of gRNA targeting the CKII phosphorylation site in the HPV-18 E7 CR2 region. gRNAs targeting the CKII phosphorylation site in HPV-18 E7 genomic region were designed using the online gRNA design platform–CRISPR MultiTargeter (http://www.multicrispr.net/). gRNA oligos were then annealed and cloned into the BbsI restriction site in pSpCas9(BB)-2A-GFP (PX458). **(B)** Surveyor assay for assessing efficiency of gRNAs. C4-1 cells were transfected with pSpCas9(BB)-2A-GFP or with the same construct plus gRNA1 or gRNA2. After 72h, cells were FACS-sorted to collect GFP-positive cells. Genomic DNA was extracted to PCR-amplify the specific region spanning E7 (414bp). Amplicons were denatured and reannealed to allow DNA heteroduplex formation, then treated with Surveyor nuclease and Surveyor enhancer (IDT) and analyzed on a 2% agarose gel. Lanes h are heteroduplex PCR products and lanes d are Surveyor nuclease digestion products for respective Cas9 and gRNA treatment constructs. **(C)** Design of single-stranded DNA oligonucleotide (ssODN) as donor template for homology-directed repair (HDR). ssODN was designed as a 100bp homology arm, flanking the predicted double strand break site to change the serine 32 to alanine (agc to gcc) and the serine 34 to alanine (tca to gca). The mutagenesis was designed to insert a unique HgaI restriction site, to allow screening of the edited clones by genomic DNA isolation, PCR amplification of the edited region and restriction digestion. **(D)** Representative agarose gel electrophoresis of Hga I digestion of 18E7 PCR amplicons from wild type and mutant clones. **(E)** Validation of the genome editing with Sanger sequencing.

To mutate the E7 CKII phospho-acceptor site in C4-1 cells, two guide RNAs were designed, aiming to generate the S32A and S34A double mutant. These are depicted in Fig 3A, together with the donor DNA sequence shown in Fig 3C, which was designed to create a new HgaI restriction site in E7 to facilitate the screening of the clones. The efficiency of the guide RNAs was first confirmed using the surveyor nuclease assay in C4-1 cells, to analyze their ability to cut the target DNA sequence. As can be seen from Fig 3B the guide RNAs efficiently targeted the relevant region of the E7 gene.

C4-1 cells were then transfected with the guide RNAs and the single-stranded donor DNA and, following an extended period of selection and single cell cloning, the cells were analysed for the S32A/S34A double mutation in the CKII phospho-acceptor site, by HgaI digestion of the 18E7 PCR amplicon and then by DNA sequencing, whereby two such clones were ultimately identified (Fig 3D and 3E).

**Fig 4:**
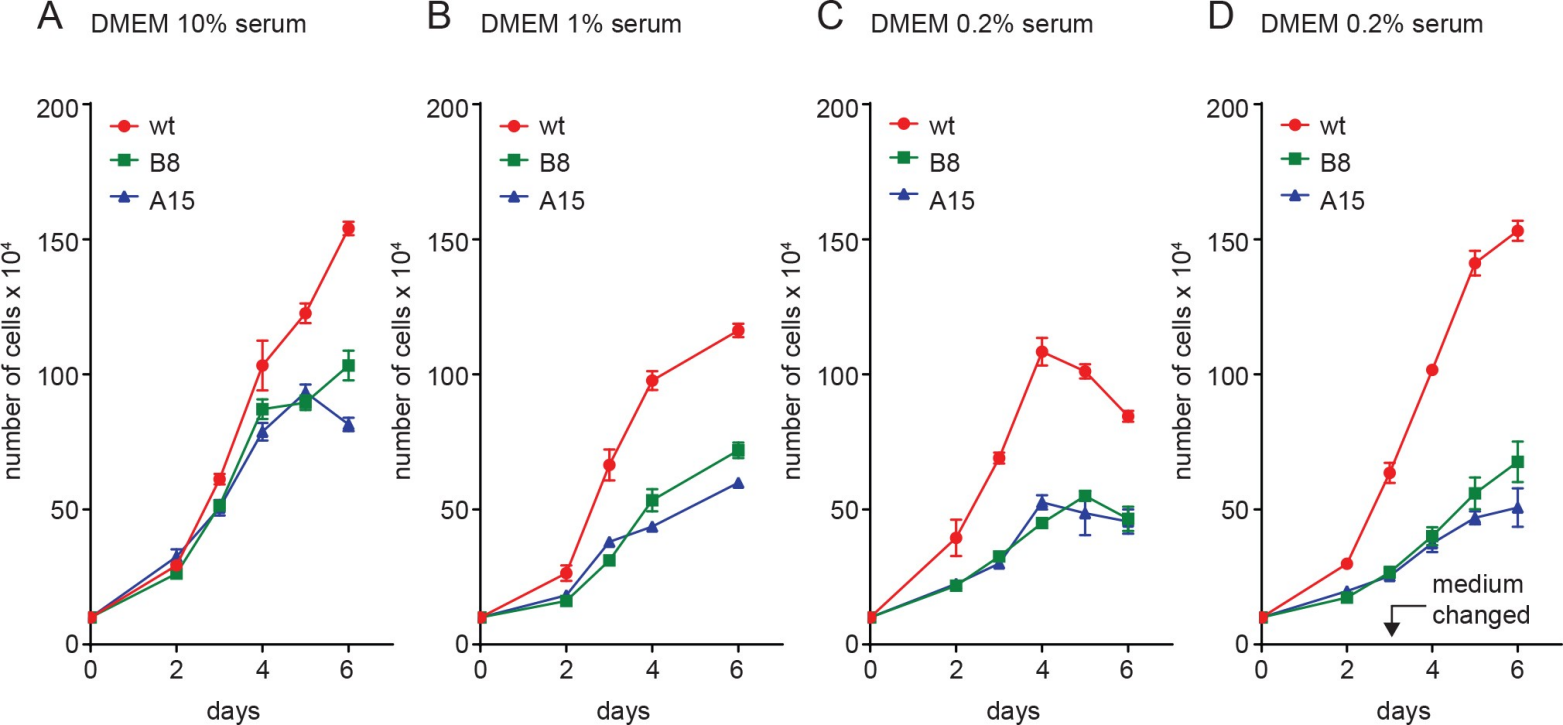
Growth of CKII site mutant cells is reduced in low nutrient conditions. **(A)** C4-1 wild type and mutant lines were seeded at 3 x 10^5^ cells per 60 mm petri dish, in DMEM with 10% fetal bovine serum and counted over a 6 day period. For determination of growth in low serum, the assay was repeated in DMEM with **(B)** 1% serum, **(C)** 0.2% serum and **(D)** 0.2% serum with medium replaced on the third day. The results show the mean of at least three independent assays and the error bars show standard deviations.

Having obtained cell lines in which the CKII HPV-18 E7 phospho-acceptor sites were mutated, we were next interested in more fully characterizing them. We first analysed their growth rates in normal and low nutrient conditions. As can be seen from Fig 4, both the mutant clones grow somewhat more slowly than the wild type cells and, furthermore, the saturation densities are also consistently lower with the mutant cell lines. This is even more apparent when the cells are grown under conditions of low serum, where in 0.2% serum there is a dramatic decrease in the growth rate in the two mutant cell lines (Fig 4C and 4D). The growth curve in 0.2% serum (Fig 4C) reaches saturation density and drops after 4 days, possibly due to nutrient deprivation and cell death. When the assay is performed replacing the medium after the third day (Fig 4D), it is clear that the wild type cells can continue to proliferate while the mutant cells have greatly reduced rates of cell proliferation. These results suggest that mutation of the CKII phospho-acceptor sites in HPV-18 E7 in C4-1 cells has a markedly deleterious effect upon the ability of the cells to proliferate.

**Fig 5:**
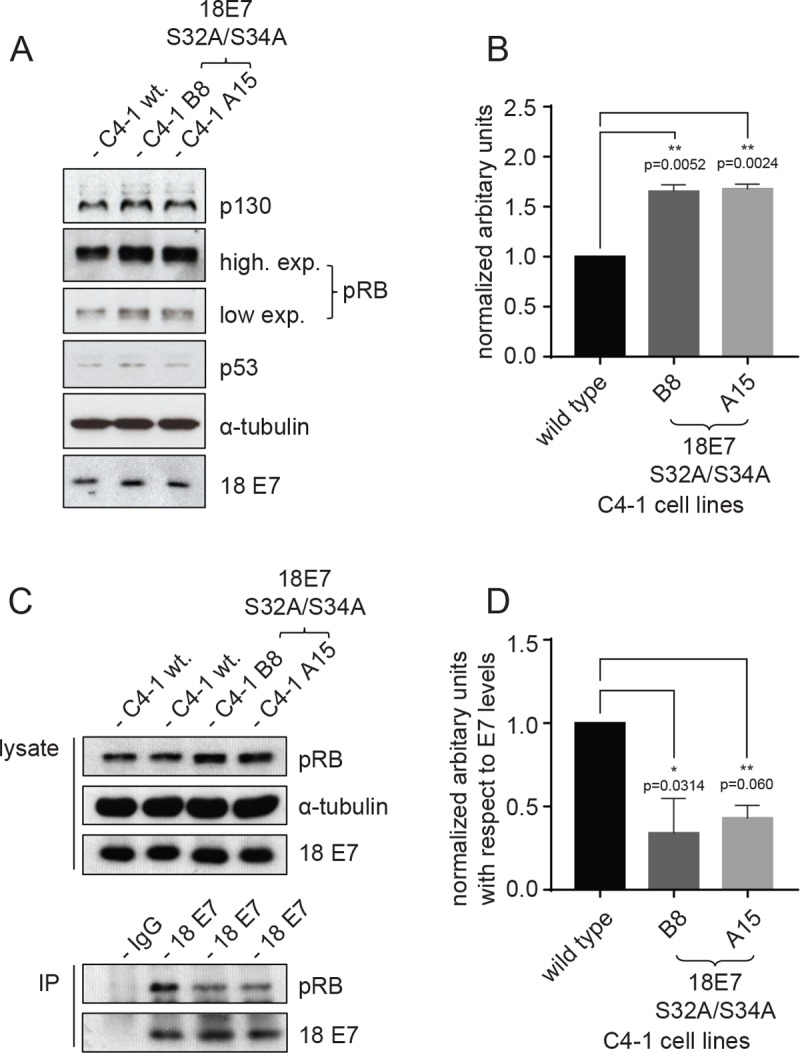
Steady-state levels of HPV-18 E7 and pocket proteins in the C4-1 cell lines. **(A)** Western blot analysis of total cell extracts obtained from the wild type and mutant C4-1 lines. These were probed for p130, pRb, p53, HPV-18 E7, and α-tubulin was used as a loading control. (**B)** Quantification of levels of pRB normalized with respect to α-tubulin shown as bars with the standard error of mean. Asterisks indicate the p-value of mean levels of pRB in mutant cells compared with wildtype cells. (**C**) Cells lysates from wildtype and CKII mutant C4-1 cells were immunoprecipitated with anti-HPV-18 E7 antibody overnight and then incubated with protein A beads for 90 minutes at 4°C. Immunoprecipitated complexes were then washed with lysis buffer and analysed by Western blotting for pRB. Mouse monoclonal anti-GFP antibody was used as control IgG. The upper panels show the protein inputs for the immunoprecipitations, with α-tubulin serving as a loading control and the results of immunoprecipitation are shown in the lower two panels. **(D)** Quantification of the levels of pRB immunoprecipitated with respect to levels of E7 shown as bars with the standard error of mean. Asterisks indicate the p-value of mean levels of pRB immunoprecipitated in mutant cells compared with wildtype cells.

Having shown that the mutant cells are less proliferative than the parental cells, we then analysed the levels of pocket proteins (pRB and p130) in these cell lines in comparison with the parental cell line. As can be seen from Fig 5, both the double mutant cell lines have slightly higher levels of pRB and p130 compared with the wild type line. Since previous studies had indicated that an intact CKII phospho-acceptor site can aid pocket protein recognition [24, 25], we also performed co-immunoprecipitation analysis between HPV-18 E7 and pRb in the wild type and mutant E7 cell lines. The results obtained are shown in Fig 5C and 5D where it can be seen that lower levels of pRb are complexed with E7 in the two mutant lines when compared with the wild type C4-1 cells. We also analysed the total levels of expression of the E7 oncoprotein and, as can also be seen from Fig 5A and 5C, mutation of the CKII phospho-acceptor site had no deleterious effects upon the levels of E7 protein. In addition, the levels of p53 were also unchanged, suggesting that the levels of E6 expression were similarly unaffected. These results demonstrate that mutation of the E7 CKII phospho-acceptor site has a modest effect upon overall levels of pocket protein expression, as would be expected from previous structural studies [17–19, 24, 25], however whether such changes are sufficient to account for the defects in cell proliferation remains to be determined.

**Fig 6:**
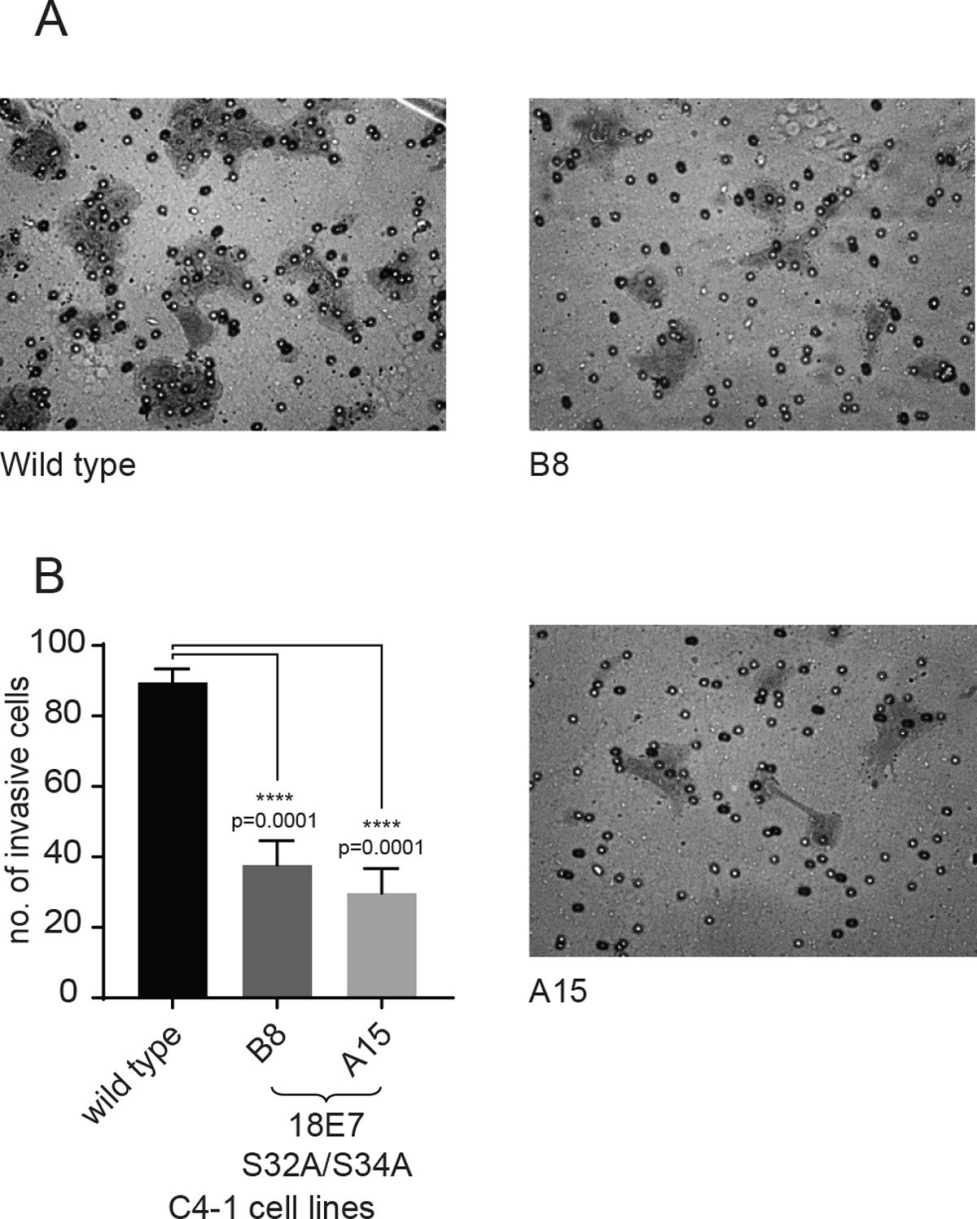
CKII site mutant C4-1 cells show defects in matrigel invasion. Equal numbers of wild type and mutant C4-1 cell lines were seeded in the upper part of Matrigel invasion chambers, in DMEM without serum. DMEM with 2% serum was used as a chemoattractant in the lower chamber. After 22h, cells that had invaded the lower chamber were fixed and stained with 0.5% Crystal Violet in 5% glutaraldehyde for 10 mins. **(A)** Representative micrographs showing invasive cells from each cell line as indicated. **(B)** Quantification of the mean number invasive cells shown as bars with the standard error of mean. Asterisks indicate the p-value of mean numbers of invasive cells, compared with the wild type cells.

We then proceeded to investigate the invasive capacity of the mutant clones using a matrigel invasion chamber assay. The cells were plated on the upper chamber without serum and a chemo-attractant stimulus was provided in the lower chamber in the form of 2% calf serum. After 22hrs the lower chamber was fixed and stained and the number of migrating cells were counted. As can be seen from Fig 6, wild type C4-1 cells are very invasive, and migrate readily through the collagen matrix. In contrast, both mutant lines show defects in their invasive potential. Thus, the mutation within the HPV-18 E7 CKII phospho-acceptor site greatly decreases the ability of the cells to invade through a collagen matrix.

**Fig 7:**
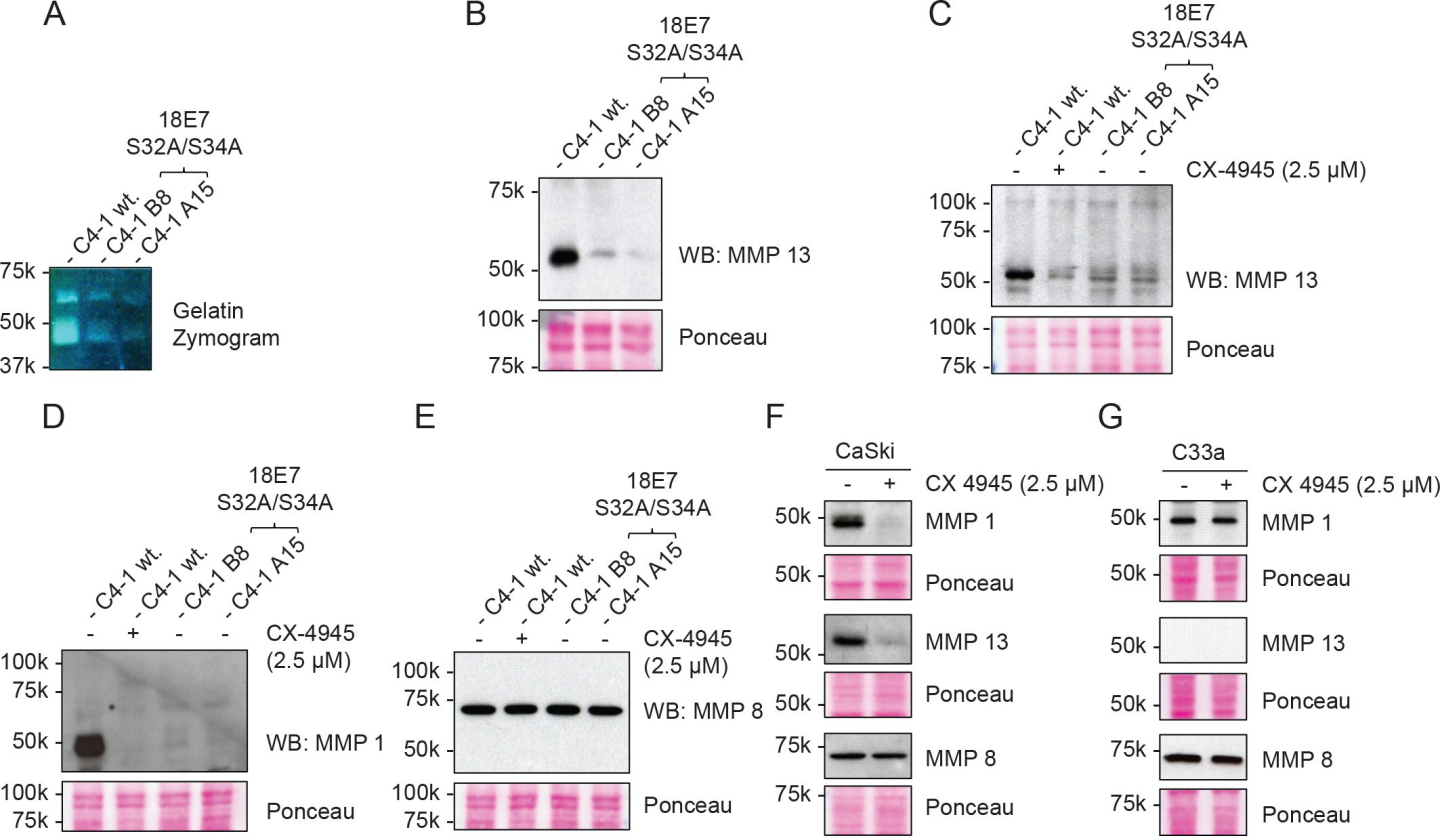
Matrix metalloproteases MMP 1 and MMP 13 are downregulated in CKII site mutant C4-1 cell lines. **(A)** Confluent wild type and CKII site mutant C4-1 cell lines were incubated in a serum-free medium for 48 hours. The conditioned media from the cell lines were concentrated and equal amounts of protein run on non-reducing 10% SDS-PAGE containing 0.1% gelatin, The gel was then renatured and incubated overnight at 37°C for gelatin hydrolysis. After incubation, the gel was stained with Coomassie Blue to visualize the bands of hydrolyzed substrate. **(B)** Equal amounts of concentrated conditioned media from the indicated cell lines were analysed by western blotting for MMP13 (upper panel). The lower panel shows the Ponceau stain of the nitrocellulose to confirm equal protein loading. Panels **(C, D, E, F, G)** Cells were processed as in panel B, but with the addition of 2.5 μM of CX-4945, as indicated, to specifically inhibit CKII activity. Conditioned media from wildtype C4-1 and mutant cells, plus CaSki and C33a cells, were then processed as before and analysed by western blotting for MMP13, MMP1 and MMP8 (upper panels), with the lower panels in each case showing the Ponceau stain of the nitrocellulose membranes.

To investigate whether the mutant cells’ defective invasion potential was related to any change in their ability to hydrolyse the extracellular matrix (ECM), we concentrated conditioned media from the mutant cells and analysed their activity by Gelatin Zymography. As can be seen in Fig 7A, conditioned media from the mutant lines are unable to hydrolyse the gelatin gel, whereas that from the wildtype cells shows significant bands of gelatin hydrolysis. We then performed a series of western blot analyses on the conditioned medium to try and determine which matrix metalloproteases might be involved. As can be seen from Fig 7B and 7D, both MMP1 and MMP13 are significantly downregulated in the two mutant clones when compared with the wild type C4-1 cells. This appears to be highly specific, since there is no change in the levels of expression of MMP8 (Fig 7E). To determine whether the defect in MMP secretion was a reflection of a slower growth rate in the clones, we repeated the assay but growth arrested the wild type C4-1 cells with mitomycin C treatment for 48hrs. Secreted MMP levels were then analysed by western blotting, and as can be seen from Supplementary S1 Fig there is no apparent difference in MMP levels in the growth arrested cells. These results indicate that the differences in MMP levels between the wild type and mutant C4-1 cells, is not due to differences in proliferation.

In order to determine whether the high levels of MMP1 and MMP13 expression are dependent upon CKII activity, we treated the wild type C4-1 cells with the highly specific CKII inhibitor CX-4945 and analysed the effects upon the protein levels of MMP1 and MMP13. As can be seen from Fig 7C and 7D, CX-4945 treatment of the wild type cells reduces the levels of MMP1 and MMP13 expression to levels similar to those seen in the two mutant lines. This suggests that active CKII phosphorylation of HPV-18 E7 is required for maintaining high levels of MMP protein expression in C4-1 cells.

**Fig 8:**
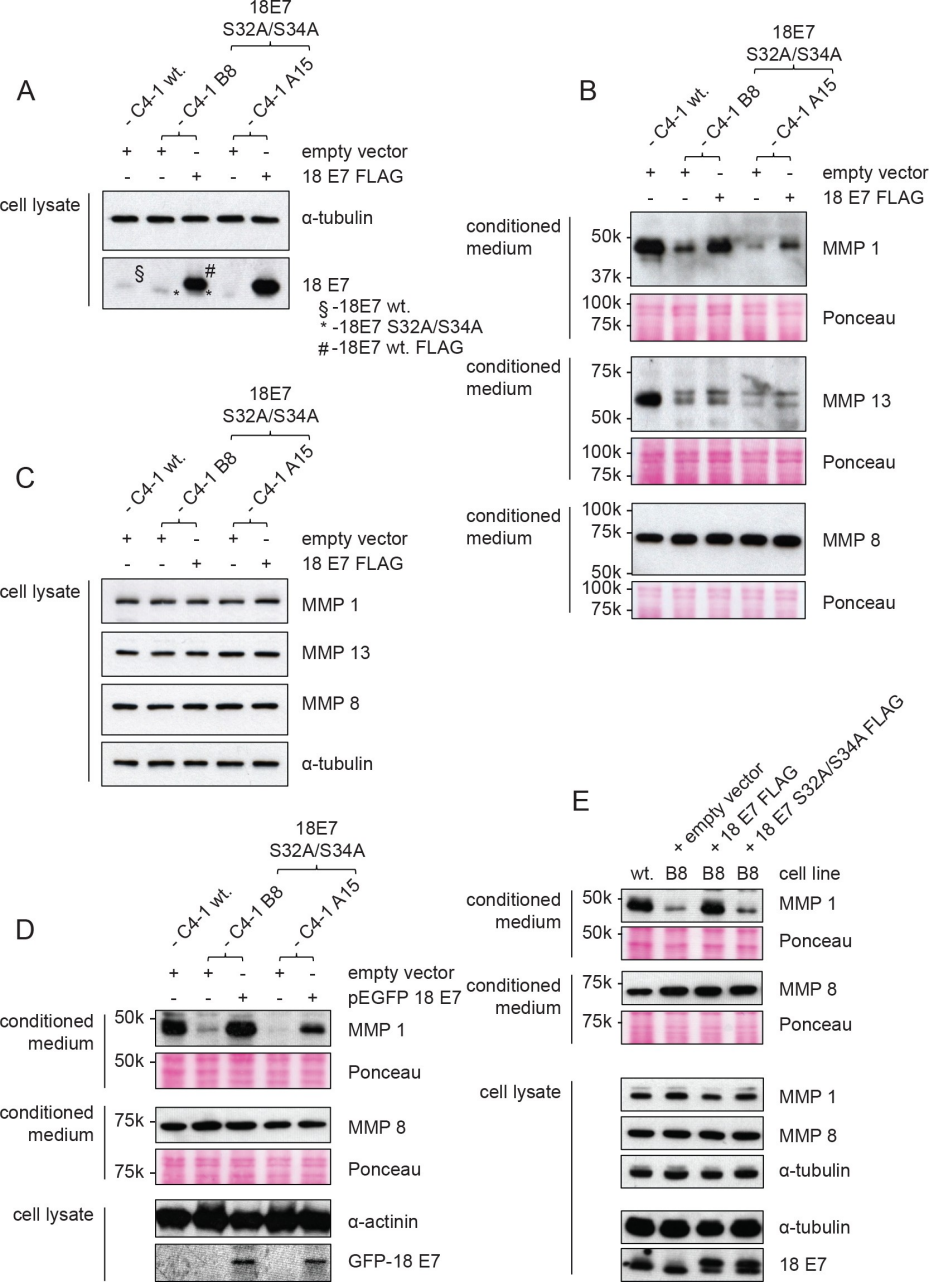
Exogenous expression of wild type HPV-18 E7 can rescue the downregulation of MMP 1 and MMP 13 in CKII site mutant lines. Wild type C4-1 cells were transfected transiently with empty pCMV vector and mutant cell lines with pCMV vector expressing FLAG-tagged wild type HPV-18 E7. **(A)** The cells were then harvested directly in 2X sample buffer and analysed by SDS-PAGE and Western blotting for levels of 18E7 and α-tubulin. The position of endogenous wild type E7 (§), the S32A/S34A mutant (*) and the wild type FLAG-tagged E7 (#) is also shown. **(B)** Concentrated conditioned medium from the cells was analysed by western blotting for MMP1, MMP13 and MMP8 (upper panels). The lower panels show the Ponceau stain of the nitrocellulose membranes to confirm equal levels of protein input. **(C)** Western blot analysis of total cell extracts for MMP1, MMP13 and MMP8 with α-tubulin used as the loading control. **(D)** C4-1 cells were transfected with pEGFP 18E7 wildtype plasmid or empty vector as indicated and selected by FACS sorting and G418 selection to obtain stable cells stably expressing GFP E7. The cells were analysed for MMP1 and MMP8 in the conditioned medium and expression of GFP 18E7 was ascertained in the cell lysate by western blotting. **(E)** C4-1 B8 CKII mutant cells were transfected with pCMV empty or pCMV 18E7 wildtype or pCMV 18E7 S32A/S34A mutant expressing plasmids as indicated and selected with G418 to obtain stable cell lines. The cells were then analysed for MMP secretion in the conditioned medium and the cell lysate by western blotting.

All the above analyses were done on the C4-1 cells, but we wished to determine whether high levels of MMP secretion were specific to active CKII in HPV-positive cells. To do this we analysed the levels of secreted MMPs in HPV-16-positive CaSki cells and HPV-negative C33a cells, in the presence and absence of CKII inhibitor. The results obtained are shown in Fig 7F and 7G and demonstrate that high levels of secreted MMP1 and MMP13 in CaSki cells are also dependent upon active CKII, whilst in C33a cells inhibition of CKII has no effect upon the levels of secreted MMPs. These results indicate a specific role for active CKII in regulating MMP levels in HPV-positive cells. To verify this, we performed a rescue experiment where we overexpressed FLAG-tagged wild type HPV-18 E7 in the two mutant lines and examined the effects on MMP levels secreted by the cells. Fig 8A shows the levels of endogenous and overexpressed HPV-18 E7 whilst, as can be seen from Fig 8B, in cells that expressed high levels of wildtype FLAG-tagged HPV-18 E7 the amounts of secreted MMP1 and MMP13 were increased, while no effect was seen on the levels of MMP8 (Fig 8B). In order to determine whether E7 affected the total levels of MMP1 expression, we also performed western blot analysis on total cell extracts derived from the wild type, mutant and E7 overexpressing cells. As can be seen from Fig 8C, the intracellular levels of MMP1 are unaffected by mutation of the E7 CKII phospho-acceptor site and remain unchanged following overexpression of wild type E7.

Since the rescue of the levels of secreted MMPs was only partial, which was most likely due to a low level (approx. 20%) of transfection efficiency, we proceeded to generate cells stably expressing wild type HPV-18 E7. To do this, the CKII mutant cells were transfected with GFP 18E7 and selected by FACS sorting and subsequent G418 selection. The pooled cells were then analysed for the levels of secreted MMPs. As can be seen from Fig 8D, both of the CKII mutant polyclonal cell lines stably express GFP 18E7. Furthermore, in these rescued cells the conditioned medium has very similar levels of secreted MMP1 to that seen in the wild type C4-1 cells. To further verify that this rescue is due to phosphorylation of stably expressed wild type HPV-18 E7, cells stably expressing a C-terminal FLAG tagged wild type HPV-18 E7 and the HPV-18 E7 S32A/S34A mutant were also obtained and analysed for MMP secretion. As can be seen from Fig 8E, despite similar levels of wild type and mutant HPV-18 E7 S32A/S34A expression, wild type levels of MMP1 secretion was only obtained in the cells expressing the wild type HPV-18 E7 protein. These results demonstrate that an intact E7 CKII phospho-acceptor site plays an essential role in ensuring high levels of MMP secretion in a HPV-18-positive cervical tumour-derived cell line.

**Fig 9:**
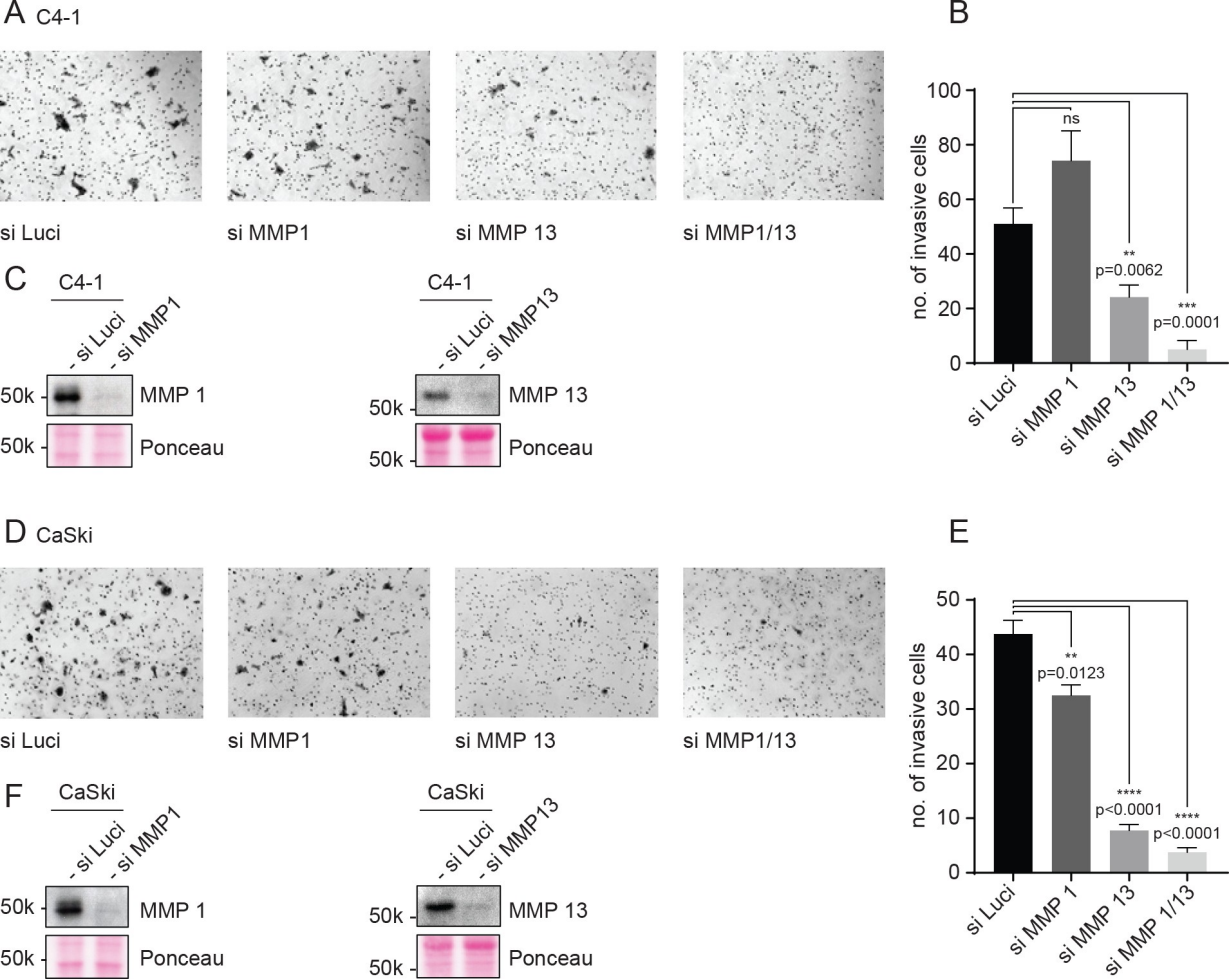
Knockdown of MMP1/13 abrogates invasion of C4-1 and CaSki cells. C4-1 and CaSki cells were transfected with indicated siRNAs. After 48 hours, equal number of cells were seeded in the upper part of Matrigel invasion chambers, in DMEM without serum. DMEM with 10% of serum was used as a chemoattractant in the lower chamber. After 22h, cells that had invaded the lower chamber were fixed and stained with 0.5% Crystal Violet in 5% glutaraldehyde for 10 mins. (**A** and **D**) Representative micrographs showing invasive cells from each siRNA treatment as indicated. (**B** and **E**) Quantification of the mean number of invasive cells per field shown as bars with the standard error of mean obtained from at least 4 separate fields. Asterisks indicate the p-value of mean numbers of invasive cells, compared with the wild type cells. (**C** and **F**) Western blot analysis of concentrated conditioned medium from indicated siRNA treatments for MMP1 and MMP13.

Having identified MMP1 and MMP13 as being upregulated in a CKII dependent manner in HPV-positive cells, we then asked if knockdown of MMP1 and MMP13 would have any deleterious affect upon the invasive ability of the wild type cells. To do this, we transfected siRNA against luciferase as control, or MMP1 or MMP13, or a combination of the two into wild type C4-1 cells, and after 48 hours pooled equal number of cells and performed a Matrigel invasion assay. As can be seen from Fig 9A–9C, there is marked decrease in invasion upon knockdown of MMP13, little effect with MMP1 knockdown alone, but a marked synergistic effect when both MMP1 and MMP13 were removed. Similar results were obtained when the assay was performed in HPV-16-positive CaSki cells (Fig 9D and 9E).

Taken together, these results demonstrate that loss of CKII phosphorylation of HPV-18 E7 in cells derived from a cervical cancer, has diverse inhibitory effects upon the ability of the cells to proliferate and to invade a collagen matrix, and the defect in the ability to invade is at least in part due to downregulation of MMP1/13.

**Fig 10:**
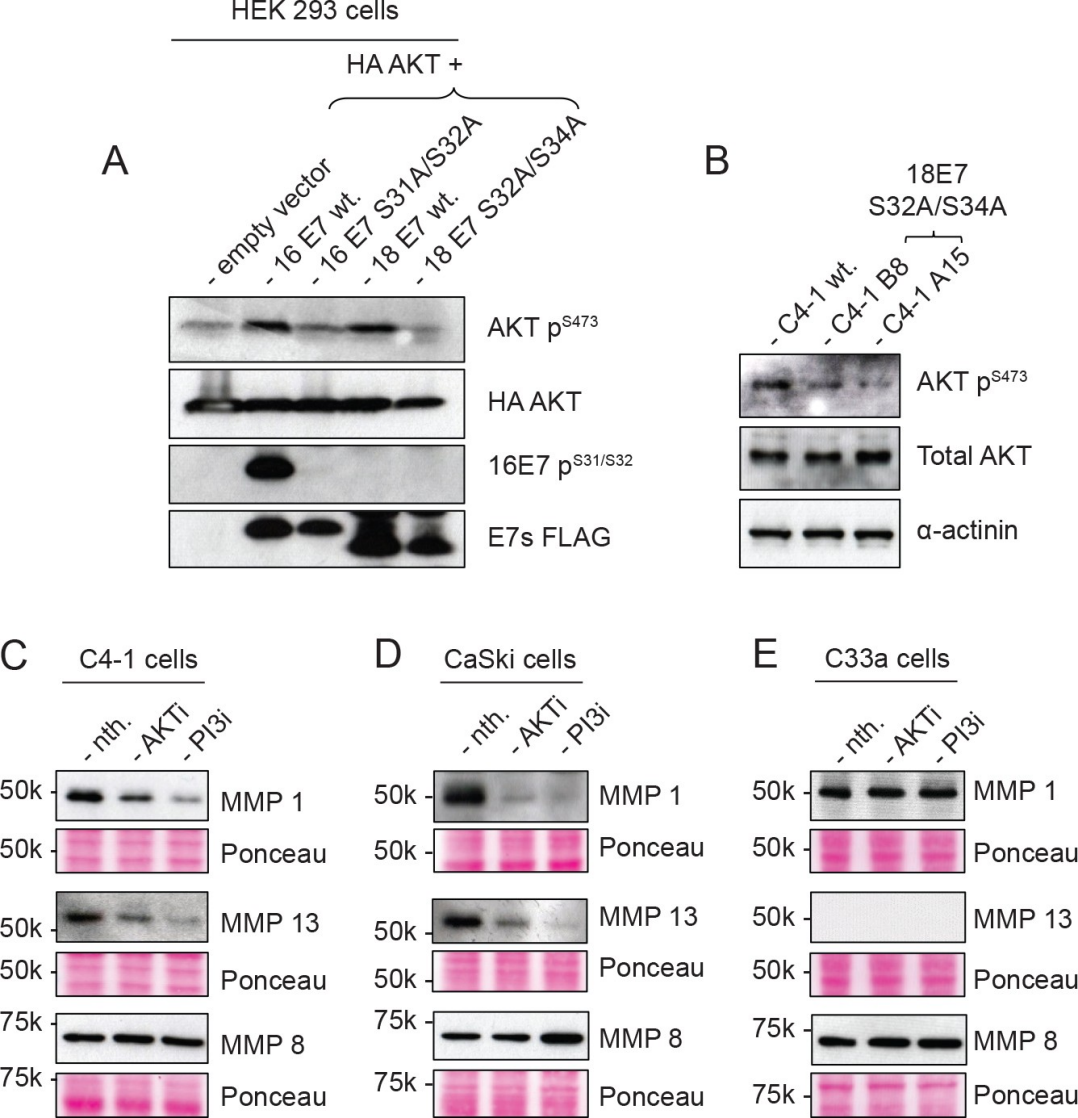
Activation of AKT is perturbed in the absence of CKII phosphorylation of E7. (**A**) HEK 293 cells were transfected with HA-AKT together with HPV-16 E7 FLAG-HA, wildtype or 16 E7 S31A/S32A, HPV-18 E7 FLAG, wildtype or 18 E7 S32A/S34A or empty vector. After 24 hours, the cells were serum starved for 16 hours and harvested directly with SDS PAGE sample buffer and analysed by Western blotting for phospho-AKT, total AKT (HA), phoshpo 16E7 and total E7 (FLAG), as indicated. (**B**) Confluent C4-1 wildtype and CKII mutant cells were serum starved for 48 hours and total levels of AKT, p-AKT, and α-actinin were analysed by Western blotting. (**C**, **D**, **E**) Confluent C4-1, CaSki and C33a cells were either treated with AKT inhibitor or PI3K inhibitor in serum free medium for 48 hours. Conditioned medium from the treatments were analysed by Western blotting for MMP1, MMP13 and MMP8. In each case the lower panels show the Ponceau stain of the nitrocellulose membranes.

We were next interested in understanding the molecular basis for this defect. Previous studies had implicated the ability of E7 to activate AKT signaling as being in part responsible for promoting invasive behavior [26]. However no studies have been done to investigate whether CKII phosphorylation of E7 can lead to activation of AKT, and whether this in turn might be responsible for the increased levels of MMP secretion. To investigate these possibilities we first analysed the ability of wild type and CKII site mutants of HPV-16 and HPV-18 E7 to activate AKT in a transient assay in HEK293 cells. In order to first verify the levels of E7 phosphorylation in these assays, we generated a phospho-specific antibody directed against the HPV-16 E7 CKII phospho-acceptor site. The characterization of this antibody is shown in Supplementary S2 Fig, where it can be seen that it only reacts against the phosphorylated form of HPV-16 E7. After 24 hours, the transfected 293 cells were serum starved for 16 hours and harvested and analysed by western blotting. As can be seen from Fig 10A, only the wild type E7 is capable of inducing AKT phosphorylation. Interestingly, the phospho-specific antibody detects the wild type E7 but fails to react against the CKII phospho-site mutant. These results confirm a significant level of E7 phosphorylation with the wild type protein, but no phosphorylation in the case of the double CKII phospho-site mutant of E7.

Having found that an intact CKII phospho-acceptor site was required for the ability of E7 to increase AKT phosphorylation, we then analysed the levels of AKT phosphorylation in the wild type and CKII phospho-site mutant C4-1 cells following serum starvation for 48 hours. The results in Fig 10B also confirm the requirement for an intact CKII phospho-acceptor site to obtain high levels of AKT phosphorylation.

These results implicate AKT signaling in HPV-positive cells as being responsible for the high levels of MMP secretion. To investigate this further we analysed the levels of MMP1 and MMP13 secretion in HPV-18-positive C4-1 cells, HPV-16-positive CaSki cells and HPV-negative C33a cells following inhibition of AKT and PI3K using the specific AKT inhibitor (124005-Calbiochem) and PI3K inhibitor (LY294002). After 48 hours the supernatants were harvested and analysed by western blotting. As can be seen from Fig 10C–10E, blocking AKT signaling in HPV-positive cells results in a marked decrease in the levels of secreted MMP1 and MMP13, whilst this has no effect in the HPV-negative cells. In agreement with results in Figs 7 and 8, which showed that CKII phosphorylation of E7 had no effect on MMP8, likewise blocking AKT signaling also had no effect on MMP8 secretion in HPV-positive cells. Taken together these results demonstrate that CKII phosphorylation of E7 promotes AKT activation, which, in turn, is responsible for increased MMP secretion and subsequent enhancement of invasive potential in HPV-positive cervical tumour-derived cells.

## Discussion

Despite extensive characterization of the different biochemical functions of the HPV E6 and E7 oncoproteins, and their respective contributions towards the development of cell transformation and malignancy, there is still very little information on the continued requirement for specific activities of E6 and E7 in cells derived from a cervical cancer. This is important, since we know there is an absolute requirement for both oncoproteins for maintaining the transformed phenotype [5–7], but which specific biochemical functions of E6 and E7 contribute towards this remains obscure. In this study we have begun to address this question by performing genome editing of the HPV-18 E7 CKII phospho-acceptor site in cervical cancer-derived C4-1 cells. We find that an intact CKII phospho-acceptor site in E7 remains important, both for optimal levels of cell proliferation and for maintaining high levels of invasive potential.

Previous studies have shown that the CKII phospho-acceptor site in HPV-16 E7 plays an important role in the HPV life cycle, and in the ability of E7 to bring about cell transformation in a variety of different experimental settings [15, 27]. Furthermore, recent studies showed that a variant HPV-16 E7, in which an extra CKII phospho-site was present at N29S, exhibited a marked increase in transforming potential [20]. Many of these activities have been linked to the effects of phosphorylation on the ability of E7 to interact with some of its cellular substrates. Indeed the acidic pocket provided by the CKII consensus motif, and the subsequent increase in negative charge following phosphorylation, have been shown to increase interaction with pocket protein family members [17–19]. One consequence of which is the enhancement of HPV-16 E7’s ability to overcome pRb-associated cell senescence [22]. Phosphorylation of HPV-16 E7 has also been shown to increase interaction with TBP [16]. HPV-18 E7 is also widely assumed to be similarly phosphorylated, however the specific phospho-acceptor site had never been formally verified [14]. CKII substrate specificity is known to be determined by multiple acidic residues located between -2 and +5 positions relative to the S/T phospho-acceptor site [28, 29]. Based on our mutational analysis we confirmed that S32/S34 are the only CKII phospho-acceptor sites in HPV-18 E7. However, S34 seems to be a more preferred CKII recognition site, although the S32A mutation appears to further enhance phosphorylation of E7 by CKII at S34.

To perform the genome editing of the CKII phospho-acceptor site, we chose C4-1 cells as these cells have only a single integrated copy of HPV-18 DNA [23]. We first confirmed previous studies demonstrating a continued requirement for E6/E7 expression for maintenance of the transformed phenotype in cells derived from a cervical cancer [5–7], as this had not formally been demonstrated in C4-1 cells. Following the design of gRNAs for targeting the two serines in E7 and subsequent transfection and selection, two mutant cell lines were eventually obtained. This demonstrates that mutation of the CKII site *per se* is not incompatible with continued cell growth and survival. However, upon more detailed analysis, it is clear that mutation of the CKII phospho-acceptor site results in quite marked effects upon the degree of cell transformation. Thus cells with a mutant CKII site in E7 grow more slowly and attain lower saturation densities than wild type E7 cells. This is even more marked when cell growth is assayed in low concentrations of calf serum, where there is a dramatic reduction in the rates of cell proliferation. None of these defects appear related to significant changes in the levels of E7 protein expression, as this was unchanged in the mutant lines. It was also unrelated to altered levels of E6 expression, as gauged by the levels of p53. One possible explanation for these slower growth rates under nutrient deprivation could be related to the lower levels of mutant E7 interaction with pocket protein family members, an effect that confirms previous studies showing a role for the acidic patch of the CKII phospho-acceptor site in pocket protein recognition [24, 25]. Indeed, these studies in C4-1 cells are the first to demonstrate unequivocally that an intact CKII phospho-acceptor site in endogenously expressed E7 does influence interaction with pocket proteins. However, whilst this may influence the rates of proliferation, it seems unlikely to be the major cause, as changes in pocket protein levels are quite small. It is tempting to suggest that it is the defect in AKT activation (see below) in the E7 CKII phospho-site mutant lines, especially under low nutrient conditions, which is primarily responsible for the slower growth rates [30].

Further analysis of the effects of the CKII mutation upon the transformed properties of the cells revealed marked alterations in the invasive potential of the mutant cell lines. Using matrigel invasion assays, we found that the mutant cells were much less invasive than the parental cells, suggesting that mutation of the E7 CKII phospho-acceptor site reduces the capacity of the cells to invade through a collagen-containing 3D matrix. This defect was not due to a generalized defect in migratory capacity, since the wild type and mutant cells showed no differences in their respective abilities to migrate in a scratch wound healing assay (S3 Fig). Instead, this points to a specific defect in migration through a collagen matrix. This therefore led us to examine any potential effects upon MMPs. Using conditioned medium from the different cell lines, we first analysed potential differences in a collagen zymograph assay. To our surprise, we found marked differences between the wild type and mutant cells lines, with the cells harbouring the CKII site mutation exhibiting much lower levels of collegenase activity. This focused our attention on a subset of MMPs, which might be responsible for this defect, and we found that two MMPs, MMP1 and MMP13 were particularly highly secreted by the wild type C4-1 cells, but were largely absent from the conditioned medium of the mutant cell lines. This defect appears to be related to CKII activity, since treatment of the wild type cells with a CKII inhibitor also resulted in a decrease in the amount of secreted MMP1 and MMP13. Similar results were also obtained in HPV-16-positive CaSki cells, where again high levels of MMP1 and MMP13 secretion were dependent upon active CKII, whilst in HPV-negative C33a cells inhibition of CKII had no effect upon MMP secretion. Obviously, confirming the link between E7 phosphorylation and MMP secretion through further genome editing in other HPV-positive cells would be a valuable confirmation, but the technical difficulties in achieving this in cells with multiple HPV copies could render such approaches inconclusive. Finally, overexpression of wild type HPV-18 E7 in the two mutant lines effectively restored the levels of secreted MMP1 to levels similar to that seen in the wild type C4-1 cells, although restoration of MMP13 was much lower. Furthermore, this rescue of MMP1 secretion was dependent upon an intact CKII phospho-acceptor site in E7, since stable expression of the S32A/S34A double mutant failed to restore the levels of MMP1 secretion. It should be emphasized that when total cell extracts were analysed for the different MMPs, there was very little difference between the different cells, suggesting that the defect was not in expression, but rather in secretion. We also confirmed that MMP13 in particular also played an essential role in promoting invasion in the matrigel invasion assay, where ablation of expression resulted in a marked decrease in invasive potential, and, where interestingly, the double knockdown of both MMP1 and MMP13 gave the most severe defect in invasive capacity.

A major question at this point was the underlying mechanism. Previous studies have shown that E7 can induce upregulation of diverse MMPs at the transcriptional level [26, 31–34], although that did not seem to be the case in the case of the mutant C4-1 cells, where total levels of MMP expression were similar to those seen in the wild type cells. Other studies had shown that E7 could also upregulate AKT activity, which in turn could affect invasive potential, [26, 35], although the potential role of the CKII phospho-acceptor site of E7 was not investigated in any of these or earlier analyses [30]. We therefore proceeded to determine whether CKII phosphorylation of E7 might impact upon the levels of AKT activation, and in turn whether this might be responsible for the high levels of MMP secretion. Using both transient overexpression and the mutant C4-1 cells, it is quite clear that an intact CKII phospho-acceptor site on both HPV-16 and HPV-18 E7 plays an important role in the activation of AKT. Most importantly however, this activation of the AKT pathway in HPV-positive cells appears to be directly involved in the increase in secretion of MMP1 and MMP13, but not of MMP8, which is unaffected by either mutation of the E7 CKII phospho-acceptor site, or by CKII or AKT inhibition. In contrast, in HPV-negative C33a cells, inhibition of AKT signaling has no effect upon the levels of secreted MMP1 and MMP13. These studies demonstrate that phosphorylation of E7 enhances AKT signaling, which in turn can increase levels of MMP secretion and invasiveness, and which might also contribute towards increased rates of cell proliferation in low nutrient conditions.

It is intriguing that these studies show a clear difference in MMP1 and 13 upregulation, but no change in MMP8. Whilst the molecular basis for this requires further experimentation it is interesting that MMP1 and MMP13 have been found to be upregulated in a variety of different tumours, including cervical, whilst MMP8 on the other hand has been associated with an anti-invasive effect in certain settings [36–41]. Our current studies are now aimed at understanding how phosphorylation of E7 might modulate MMP secretion, and our focus is upon potential interaction with endocytic transport pathways.

In summary, these studies demonstrate that the activity of the E7 CKII phospho-acceptor site remains functional and relevant in cells derived from cervical cancers and indicates that the CKII phospho-acceptor site remains a very attractive candidate target for therapeutic intervention in HPV-induced malignancy.

## Materials and methods

### Cells

The C4-1 cell line was obtained from the American Type Culture Collection (ATCC) and maintained in Dulbecco’s modified Eagle’s medium (DMEM), supplemented with 10% fetal bovine serum, glutamine (300 μg/ml), and penicillin-streptomycin (100 U/ml).

### Inhibitors and chemical reagents

The inhibitors and chemicals used for the experiments are as follows: 2.5 μM Silmitasertib (CX-4945), 5μM AKT inhibitor (124005-Calbiochem), 15μg/mL PI3K inhibitor (LY294002) and 10μg/mL Mitomycin C.

### Plasmid constructs

The GST 18 E7 S32A/S34A was synthesized by the Gene Art Gene Synthesis protocol (Invitrogen) and cloned into the BamHI/EcoRI sites of the pGEX2T for GST fusion protein expression. The wild-type pGEX 18E7 plasmid was a kind gift from Karl Münger. The same plasmid was used to generate pGEX 18 E7 S32A and pGEX 18E7 S34A using a modification of the QuickChange site-directed mutagenesis system (Stratagene), according to the manufacturer’s instructions, with the following primers: S32A forward primer 5’-TGTCACGAGCAATTAGCGGACTCAGAGGAAGAA-3’ and S32A reverse primer 5’- TTCTTCCTCTGAGTCCGCTAATTGCTCGTGACA-3’; S34A forward primer 5’-GAGCAATTAAGCGACGCAGAGGAAGAAAACGAT-3’ and S34A reverse primer 5’-ATCGTTTTCTTCCTCTGCGTCGCTTAATTGCTC-3’. pSpCas9(BB)-2A-GFP (PX458) was purchased from Addgene.

The C-terminal FLAG-tagged HPV-18 E7 wild type and S32A/S34A CMV plasmid construct was generated by amplification and sub-cloning of the E7 coding sequences from the wild-type pGEX 18 E7 and pGEX 18 E7 S32A/S34A constructs as templates respectively into the pCMV Neo Bam (XhoI) empty vector (kindly provided by J. Mymryk). The primers used were the following: HPV-18 E7 forward primer 5’-CGACGGATCCGATTCGAGACCATGCATGGACCT-3’ and reverse primer 5’-GCATCTCGAGCTACTTGTCATCGTCGTCCTTGTAGTCCTGCTGGGATGC-3’. The amplified sequences were visualized by agarose gel electrophoresis, purified by using a QIAquick gel extraction kit (Qiagen), digested with BamHI and XhoI restriction enzymes, and ligated into pCMV Neo Bam (XhoI).

The C-terminal FLAG/HA-tagged pCMV HPV-16 E7 plasmid was a kind gift from Karl Münger [42]. The same plasmid was used to generate pCMV HPV-16 E7 S31A/S32A using the forward primer 5’-CTCTACTGTTATGAGCAATTAAATGACGCCGCAGAGGAGGA-3’ and the reverse primer 5’-TCATCCTCCTCCTCTGCGGCGTCATTTAATTGCTCATAACA-3’ using the QuickChange site-directed mutagenesis system (Stratagene) as described previously. The pEGFP 18 E7 was generated by PCR amplification and sub-cloning of E7 coding sequences from wildtype CMV 18E7 FLAG plasmid as template using the forward primer 5’- ATGCGAATTCATGCATGGA-3’ and the reverse primer 5’- GCATTCTAGATT ACTGCTG-3’ into EcoRI and XbaI restriction sites of the pCANmyc-EGFP 16E7 (JMB-04093) plasmid (kindly provided by J. Mymryk). pEGFP-C1 empty plasmid was from Invitrogen. The HA-PKB expression plasmid has been described previously [30].

### *In vitro* phosphorylation assay

Purified GST fusion proteins were incubated with CKII enzyme (NEB) in 20 μl kinase buffer (20 mM Tris-HCI [pH 7.5], 5 mM MnCl_2_) in the presence of 50 μCi [γ-32P] ATP (2,000 Ci/mmol)) for 15 min at 30°C. After extensive washing with kinase wash buffer (20 mM Tris-HCI [pH 7.5], 5 mM MnCl_2_, 0.1% NP-40), GST fusion proteins were subjected to SDS-PAGE and autoradiographic analysis [16].

### siRNA transfections

For transfection of siRNAs, C4-1 cells were seeded at 2 x 10^5^ cells in 60 mm petri plates and grown overnight in a humidified CO_2_ incubator. Lipofectamine RNAiMAX (Invitrogen) was used to transfect siRNAs against luciferase, HPV-18 E6 (5’-CUCUGUGUAUGGAGACACAT-3’), HPV-18 E6/E7 (5’-CAUUUACCAGCCCGACGAG-3’), MMP1 (Dharmacon) and MMP13 (Dharmacon) according to the manufacturer’s instructions.

### Design of gRNAs, homology directed repair (HDR) template and screening strategy of genome edited clones

A couple of gRNAs targeting CKII phosphorylation site in HPV 18 E7 genomic region was designed using online gRNA design platform–CRISPR MultiTargeter (http://www.multicrispr.net/). gRNA oligos (gRNA1–5’-CACCGCGAGCAATTAAGCGACTCAG-3’ and 5’-AAACCTGAGTCGCTTAATTGCTCGC-3’; gRNA2–5’-CACCGTTAATTGCTCGTGACATAGA-3’ and 5’-AAACTCTATGTCACGAGCAATTAAC-3’) were then annealed and cloned into the BbsI restriction site in pSpCas9(BB)-2A-GFP (PX458).

Single stranded DNA oligonucleotide (ssODN) as donor template for homology-directed repair (HDR) was designed as 100bp homology arm flanking the predicted double strand break site to abolish phosphorylation, substituting serine 32 with alanine (agc to gcc) and serine 34 with alanine (tca to gca). The designed mutagenesis also included a unique Hga I restriction site, to allow screening of the edited clones by genomic DNA isolation, PCR amplification of edited region and Hga I restriction digestion.

### gRNA transfection, FACS sorting and surveyor assay

C4-1 cells were transfected with pSpCas9(BB)-2A-GFP or with the same construct with gRNA1 or gRNA2 using the Amaxa Cell Line Nucleofector Kit C according to the manufacturer’s instructions. Transfected cells were incubated for 72 hrs in a humidified CO_2_ incubator and then harvested with trypsin, washed once with PBS and resuspended in 5mM EDTA [pH 8.0] in PBS for FACS (BD FACSAria II) sorting. GFP-positive cells were collected and genomic DNA was isolated using the Wizard Genomic DNA Purification Kit (Promega) according to the manufacturer’s instructions. HPV 18 E7 ORF was then amplified (414 bp) using forward primer 5’-CCAACGACGCAGAGAAACAC-3’ and reverse primer 5’-AAACCAGCCGTTACAACCCG-3’. Amplicons were then denatured and reannealed as follows: 95°C for 10 min, 95°C to 85°C for 1 min, 85°C to 75°C for 1 min and so on for every 10°C decrease in temperature for 1 min and finally a hold at 25°C for 1 min using a thermal cycler to allow DNA heteroduplex formation. The heteroduplexes were then treated with Surveyor nuclease and Surveyor enhancer (IDT) and analyzed on a 2% agarose gel. The cleavage intensity of the gRNAs was calculated by analyzing band intensities using the ImageJ program. The indel percentage caused by respective gRNA was then calculated as described by Ran et al. [43].

### Isolation, screening and verification of C4-1 mutant lines

C4-1 cell lines were transfected as described above with pSpCas9(BB)-2A-GFP gRNA1 together with ssODN HDR template and incubated in a humidified CO_2_ incubator. Seventy-two hours after transfection, the top 10% GFP-positive cells were sorted as described above and seeded in limiting dilution in 100 mm diameter petri dishes for isolation of single cell clones using cloning chambers or 96 well tissue culture plates. Genomic DNA extraction of the single cell colonies and PCR amplification of the E7 ORF was performed as before. Then, individual PCR amplicons were digested with Hga I restriction enzyme and analyzed on a 2% agarose gel. Clones positive for Hga I restriction digestion were further verified by Sanger sequencing for genome editing at the CK II site.

### Determination of growth kinetics

C4-1 wild type and mutant lines were seeded at 3 x 10^5^ cells in a 60 mm petri dishes in DMEM with 10% fetal bovine serum. Cells were harvested with trypsin, dispersed and counted using a hemocytometer every single day or two. For determination of growth in low serum, the experiment was performed independently in DMEM with 1% and 0.2% fetal bovine serum. Cell counts were analyzed using Graphpad Prism 7.0 to generate a growth curve.

### Antibodies, western blotting and co-immunoprecipitation

Following antibodies were used for western blotting. Mouse monoclonal anti-p53 antibody (DO-1), rabbit polyclonal anti-p130 antibody (C-20), mouse monoclonal anti-HPV18 E7 antibody (F-7), mouse monoclonal anti-MMP-1 antibody (SB12e), mouse monoclonal anti-MMP-8 (B-1), mouse monoclonal anti-MMP13 (C-3), mouse monoclonal anti-α-actinin antibody (H-2), mouse monoclonal anti-GFP Antibody (B-2) from Santa Cruz Biotechnology; mouse monoclonal anti-Rb (G3-245; BD Pharmingen), mouse monoclonal anti-α-tubulin, mouse monoclonal anti-HA-peroxidase (clone HA-7), mouse monoclonal anti-FLAG-M2-peroxidase from Sigma, mouse monoclonal anti-18E6 antibody (N-terminus #399; Arbor Vita Corporation), rabbit Akt antibody #9272, rabbit anti phospho-Akt (Ser473) antibody #9271 from Cell Signaling. HPV-16 E7 pS31/S32 peptide antibody was generated by Eurogentec (peptide sequence: C+EQLND-S(PO_3_H_2_)S(PO_3_H_2_)-EEED and validated by ELISA and Western blotting. Secondary anti-rabbit HRP and anti-mouse HRP antibodies were obtained from Dako.

For Western blotting, total cell extracts were obtained by lysing the cells directly in 2X SDS-PAGE sample buffer, and were then separated by SDS-PAGE and blotted on 0.22-μm nitrocellulose membrane. Membranes were blocked in 5% non-fat dry milk in TBST (20mM Tris-HCl pH 7.5, 150 mM NaCl, 0.1% Tween-20) for 1 h and probed with appropriate primary and secondary antibodies. The blots were then developed using the ECL Western blotting detection reagent (GE Healthcare) according to the manufacturer’s instructions.

For detection of extracellular MMPs, confluent cells were cultured in serum free medium for 48 hours and conditioned media from cell lines were concentrated 10 times using Amicon Ultra-4 centrifugal devices and equal amounts of proteins were mixed with 2X SDS-PAGE sample buffer and processed as described previously for Western blotting.

For co-immunoprecipitation, wild type and CKII phospho-acceptor site mutant C4-1 cells were harvested using lysis buffer (50mM HEPES pH7.4, 150mM NaCl, 1mMMgCl_2_, 1mM NaF, 1% Triton-x-100, protease inhibitor cocktail I [Calbiochem]) and incubated with 1μg of mouse monoclonal HPV-18 E7 antibody overnight at 4°C. Mouse monoclonal IgG antibody against GFP was used as a control. After incubation, immune complexes were incubated further with Protein A beads (Immobilized protein A 300, Repligen) for 90 minutes at 4°C, followed by four washes in the lysis buffer. Immunoprecipitates were then run on SDS PAGE and analysed by Western blotting.

### Matrigel invasion assays

Matrigel Invasion Assays were performed as described previously [44]. Briefly, Matrigel invasion chambers (Corning BioCoat Matrigel Invasion Chamber) were brought to room temperature and rehydrated with DMEM without serum for 2 hours in a humidified CO_2_ incubator. Wild type and mutant C4-1 cells were seeded at 1×10^5^ cells in 200 μl of growth medium into the upper chamber. After allowing the cells to attach for 1 h, the medium in the upper chamber was replaced with DMEM without serum, while DMEM with 2% serum was added to the lower chamber as chemoattractant. After 22 hours, DMEM and any cells remaining in the upper chamber were removed by wiping with a cotton swab. Cells that had invaded the lower chamber were then fixed and stained with 0.5% Crystal Violet in 5% glutaraldehyde for 10 mins. After washing the excess stain with distilled water, the membrane was removed from the insert housing and placed on a microscope slide for imaging and analysis using a transmitted light microscope at 20X magnification. At least three fields per membrane were counted for each cell line. Invaded cell counts were analyzed using Graphpad Prism 7.0.

### Gelatin Zymography

Wild type and mutant C4-1 cell lines were incubated in a serum-free medium for 48 hours and conditioned media from the cell lines were concentrated 10 times using Amicon Ultra-4 centrifugal devices. Equal amounts of proteins were mixed with non-reducing sample buffer (125mM Tris-HCl pH 6.8, 4% SDS, 20% glycerol and 0.01% bromophenol blue) and run on a 10% SDS-PAGE containing 0.1% gelatin. The gel was renatured by washing with 2.5% Triton X-100 in 50mM Tris pH 7.4, 5mM CaCl_2_ and 1 μM ZnCl_2_ for 1 hr. After rinsing briefly with deionized water, the gel was incubated overnight at 37°C in 1% Triton X-100, 50mM Tris pH 7.4, 5mM CaCl_2_ and 1 μM ZnCl_2_ and stained with Coomassie staining solution (0.5% Coomassie G250, 40% Methanol, 10% Acetic acid and 50% deionized water) for 1 hr and de-stained in 40% Methanol, 10% Acetic acid and 50% deionized water, until clear bands of hydrolyzed substrate were visualized.

## Supporting information

S1 Fig Higher levels of MMP1 and MMP13 in condition medium of C4-1 wild type is independent of cell proliferation. Confluent cells as indicated were changed to serum free medium and incubated for 48 hours. Ten μg/mL of mitomycin C was added to one of the wild type C4-1 cells to inhibit cell proliferation. After 48 hours conditioned media from the cells were harvested, concentrated and analyzed by SDS PAGE and Western blotting to detect levels of indicated MMPs.
(TIF)

S2 Fig Validation of HPV-16 E7 phospho-specific antibody. Purified GST fusion proteins, as indicated, were incubated either with or without CKII enzyme (NEB) in kinase buffer in the presence of ATP for 15 min at 30°C. After extensive washing with kinase wash buffer, GST fusion proteins were analysed by Western blotting for HPV-16 E7 phospho-specific antibody. Levels of GST fusion proteins are shown by Ponceau staining of the nitrocellulose membrane.
(TIF)

S3 Fig Scratch wound healing assay for migratory abilities of C4-1 cells. Confluent wild type and CKII mutant C4-1 cells were scratched with a sterile Artline p2 pipette tip. The cells were washed twice with PBS and photographed immediately and after 24 hours. The decrease in area of the scratch was analysed and quantified using the Image J and Prism programs, is shown as bars with standard error of mean.
(TIF)
