# Supplementary figures and images for: The HPV-18 E7 CKII phospho acceptor site is required for maintaining the transformed phenotype of cervical tumour-derived cells
Source: PLoS Pathog. 2019 May 22;15(5):e1007769. doi: 10.1371/journal.ppat.1007769 (PMC6530875; doi:10.1371/journal.ppat.1007769)

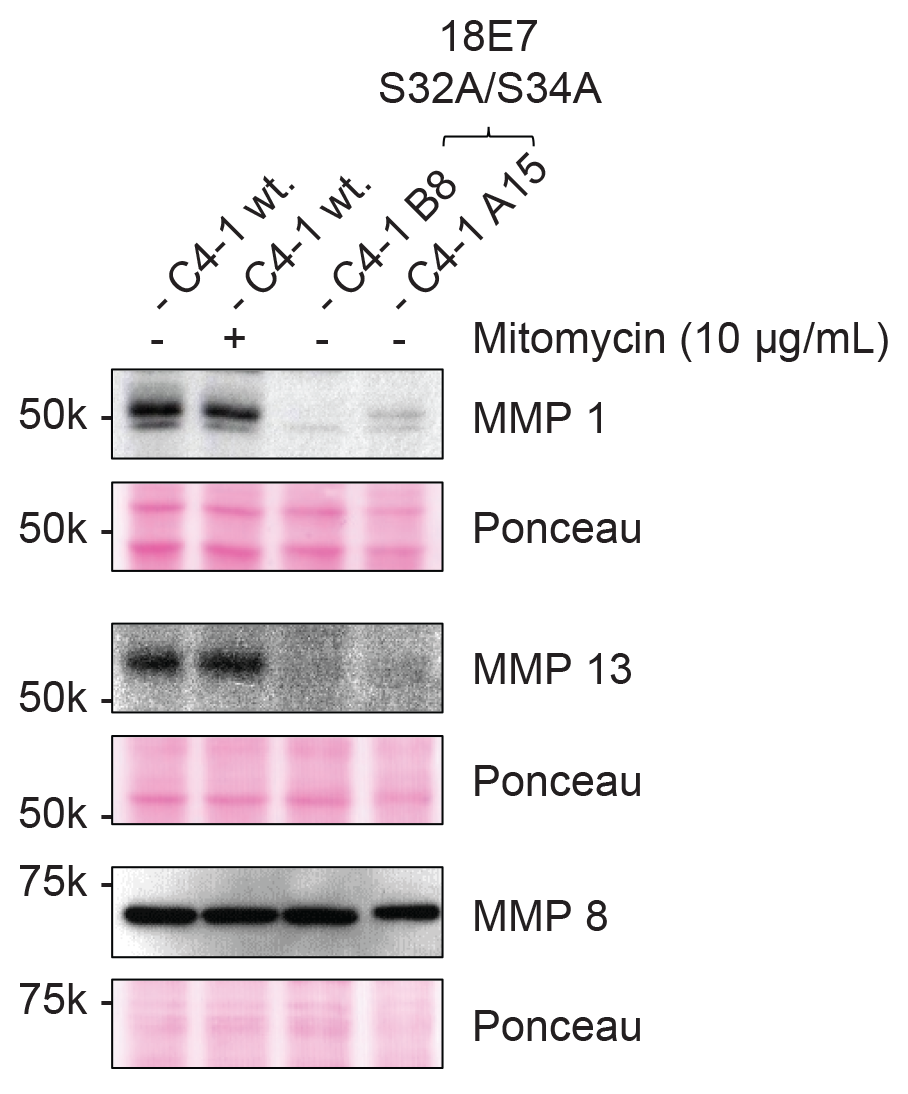

Supplement: S1 Fig — Confluent cells as indicated were changed to serum free medium and incubated for 48 hours. Ten μg/mL of mitomycin C was added to one of the wild type C4-1 cells to inhibit cell proliferation. After 48 hours conditioned media from the cells were harvested, concentrated and analyzed by SDS PAGE and Western blotting to detect levels of indicated MMPs. (TIF) [file ppat.1007769.s001.tif]

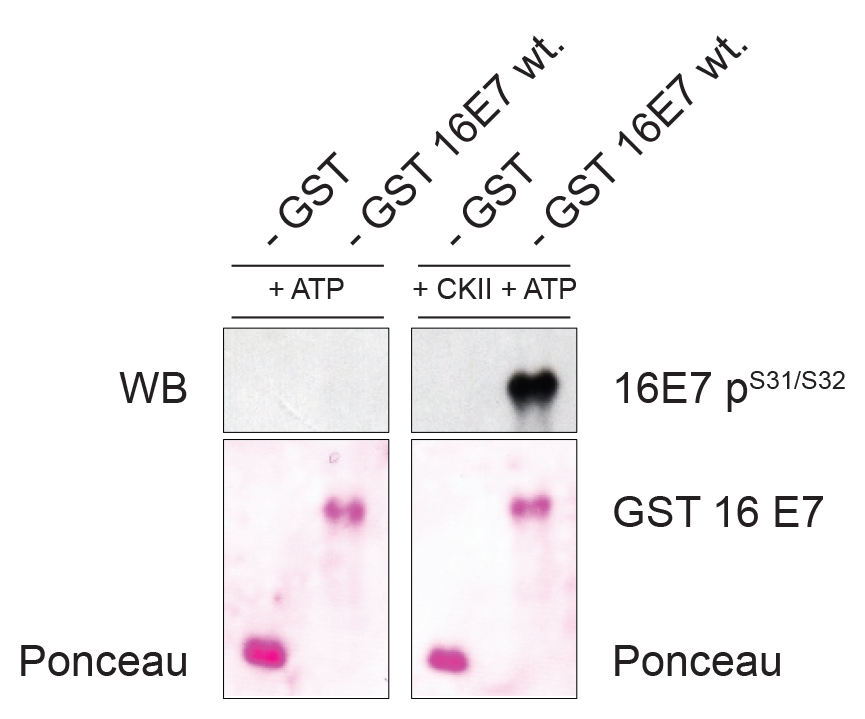

Supplement: S2 Fig — Purified GST fusion proteins, as indicated, were incubated either with or without CKII enzyme (NEB) in kinase buffer in the presence of ATP for 15 min at 30°C. After extensive washing with kinase wash buffer, GST fusion proteins were analysed by Western blotting for HPV-16 E7 phospho-specific antibody. Levels of GST fusion proteins are shown by Ponceau staining of the nitrocellulose membrane. (TIF) [file ppat.1007769.s002.tif]

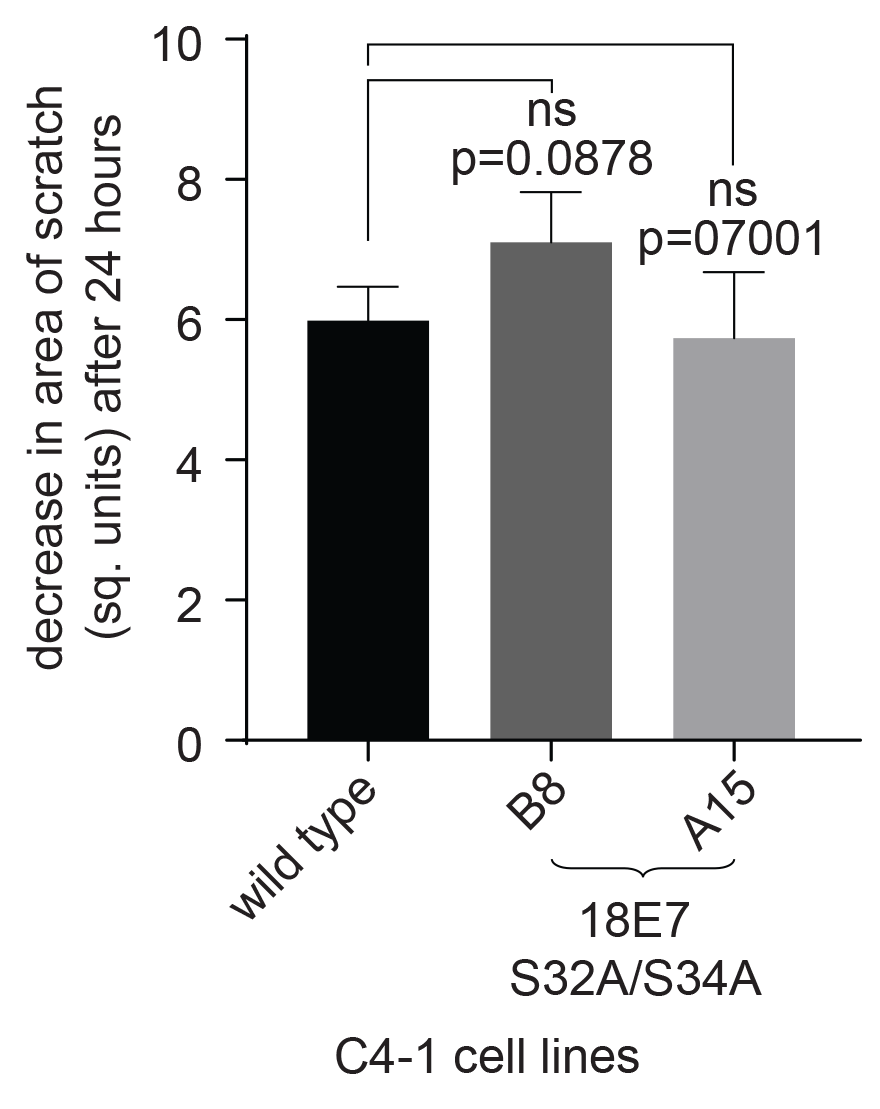

Supplement: S3 Fig — Confluent wild type and CKII mutant C4-1 cells were scratched with a sterile Artline p2 pipette tip. The cells were washed twice with PBS and photographed immediately and after 24 hours. The decrease in area of the scratch was analysed and quantified using the Image J and Prism programs, is shown as bars with standard error of mean. (TIF) [file ppat.1007769.s003.tif]
